# WASH infrastructure and practices in primary health care clinics in the rural Vhembe District municipality in South Africa

**DOI:** 10.1186/s12875-020-01346-z

**Published:** 2021-01-04

**Authors:** N. Potgieter, N. T. Banda, P. J. Becker, A. N. Traore-Hoffman

**Affiliations:** 1 Department of Microbiology, University of Venda, Thohoyandou, South Africa; 2 Research Office, Faculty of Health Sciences, University of Pretoria, Pretoria, South Africa

**Keywords:** *Escherichia coli*, Hygiene, Public health care facilities, Sanitation, Water supply and quality

## Abstract

**Background:**

South Africa has unique and diverse social and economic factors that have an impact on the provision of basic water, sanitation, hygiene and waste management infrastructure and practices at health care facilities in ensuring patient safety and prevent the spread of diseases.

**Methods:**

The aim of this study was to evaluate water, sanitation and hygiene access and standards at 50 government owned public health care clinics in the rural region of the Vhembe district of South Africa during 2016/2017, using self-observation, an observation checklist, record reviews and interviews with clinic managers. Water quality from all available water sources on the clinic compound was analysed for Total coliform and *E. coli* counts using the Colilert Quanti-tray/2000 system. The prevalence of pathogenic diarrhea causing *E. coli* strains was established using multiplex-Polymerase Chain Reaction.

**Results:**

The health care clinics in the Vhembe District generally complied with the basic WASH services guidelines according to the World Health Organisation. Although 80% of the clinics used borehole water which is classified as an improved water source, microbiological assessment showed that 38% inside taps and 64% outside taps from the clinic compounds had TC counts higher than guideline limits for safe drinking. Similarly, EC counts above the guideline limit for safe drinking water were detected in 17% inside taps and 32% outside taps from the clinic compounds. Pathogenic EAEC, EPEC, ETEC and EHEC strains were isolated in the collected water samples. Although improved sanitation infrastructures were present in most of the clinics, the sanitary conditions of these toilets were not up to standard. Waste systems were not adequately managed. A total of 90% of the clinics had hand washing basins, while only 61% of the clinics had soap present and only 64% of the clinics had adequate signs and posters reminding the staff, care givers and patients to wash their hands.

**Conclusions:**

Various WASH aspects within the primary health care system in South Africa needs to be improved and corrected. A more rigorous system that is inclusive of all role players in the WASH sectors, with regular monitoring and training sessions, should be used.

## Background

The global action plan for WASH (water, sanitation and hygiene) in health care facilities state that by 2030, every health care facility in every setting must have safely managed, reliable water, sanitation and hygiene facilities and practices to meet staff and patient needs in order to provide quality, safe people-centred care [1, 2]. However, several reports on WASH services/infrastructure and practices in health care facilities have shown disparities in the African region and other developing countries [3–9]. The practice of poor WASH in health care facilities can result in numerous consequences. It is estimated that health care associated infections (HAI) affect several millions of people every year with an estimated 15% of patients developing one or more infections during their visit and stay at health care facilities [10]. Improved WASH conditions at health care facilities establishes trust and encourage mothers to seek prenatal care and deliver in facilities rather than at home [11, 12]. It is therefore vital to improve WASH services in primary health care facilities especially when looking at birth episodes and combating antimicrobial resistance [13, 14].

The WHO guideline definition for basic water services at health care clinics, state that the main water source must be an improved water source, located on the premises and the water should be available continuously [15]. Water is an essential element in the day-to-day activities of the workers in health care facilities. Enough water is needed for drinking, cooking, hand hygiene, showering and/or bathing, for cleaning rooms, beds, floors, toilets, sheets and laundry, and to reduce the risk of infections [16]. During the Ebola epidemic in West Africa during 2014–2016, the lack of water at health care facilities was a logistic challenge to contain the outbreak [17]. A study by Majuru et al., [18] has shown that intermittent or unreliable water supplies are associated with high number of gastrointestinal diseases in sub-Saharan Africa. Saxena et al., [19] and Adebe et al., [20] have both shown that health is compromised if ill patients visit a health care facility with unsafe water. In order to assess the microbiological safety of drinking water sources, indicator organisms such as Total coliform (TE) bacteria and *Escherichia coli* (EC) bacteria are used. Total coliform bacteria include species like the *Escherichia* spp., *Klebsiella* spp., *Enterobacter* spp. and *Citrobacter* spp. (to mention a few) which have been associated with disease outbreaks and infections globally [21, 22]. The presence of the EC bacteria in drinking water indicates a recent faecal-oral contamination since the organism can only survive for limited periods outside the host and can either be present due to unprotected water supply/source to the facility, or due to the improper faecal disposal in the facility that contaminates the water [23]. Pathogenic EC strains do not all carry the same public health profile, but they all have the potential to cause disease (most notably diarrhoea) and continue to present challenges to human health and cause morbidity and mortality worldwide [24, 25].

The WHO guideline definition for basic sanitation services in health care facilities recommend that sanitation services should be improved and usable (available, private and functional) facilities with dedicated toilets to staff, sex separated toilets with menstrual hygiene facilities and toilets for use by people with limited mobility [15]. As a basic human right, sanitation services in health care facilities are crucial for the delivery of high-quality care for improved health, welfare and dignity of both patients and staff. In the absence of proper toilets, diseases can spread and therefore the sanitary management of excreta is vital to stop faecally transmitted pathogens from contaminating the environment around (inside and outside) the health facility [15].

The WHO definition for basic hygiene services state that the hand hygiene facilities must be available and functional at one or more points of care (points where care or treatment is delivered) and within 5 m from the toilet [15]. The prevention and control of infections and spread of germs in health care facilities are done through active hand hygiene measures practicing by staff and patients as well as the provision of hand washing stations with soap for patients and other [15, 26]. However, according to WHO [27], one in every four PHC facilities lack basic water services and many people are served by facilities without hand washing facilities. Musu et al., [28] have shown that organisms responsible for health care associated infections are frequently carried on the hands of health care workers. A study by Erasmus et al., [29] has shown that compliance with hand washing standards amongst health care providers is often low and may lead to disease transmission. Labi et al., [30] have shown that hand washing compliance amongst health care workers improve through proper and continued education, regular hygiene audits and feedback together with the provision of essential supplies like soap and disinfection solutions, displays of hand hygiene posters, visible and clear hand hygiene instructions and positive role modelling by senior staff and colleagues.

The WHO [15] guidelines recommend that wastes which is either infectious, chemically hazardous or radioactive must be managed in a suitable manner to prevent unsafe exposure to health care workers, patients, visitors, waste handlers and the public. Most wastes produced in health care facilities are not hazardous and can be disposed of along with general solid wastes [31, 32]. In South Africa waste management is multi-sectoral and involves not only the Department of Health (DOH) but also the Department of Environmental affairs (DEA). The DEA has developed a policy that describes the guidelines for health care risk waste management (HCRW) and the standards for equipment to be used in it. From this guideline, the DOH in each province, extracts their own guideline document with clear description on every point of the management process within health care facilities [33]. The Health Professionals Council of South Africa [34] has also developed a HCWR management booklet with guidelines on various subcategories of wastes with clear role definitions for each health worker in the HCRW management chain with specific responsibilities clearly described.

The objective of this study was therefore to assess WASH infrastructure and practices in primary health care clinics in the Vhembe District in the Limpopo Province of South Africa.

## Methods

### Study design and site selection

**Fig. 1:**
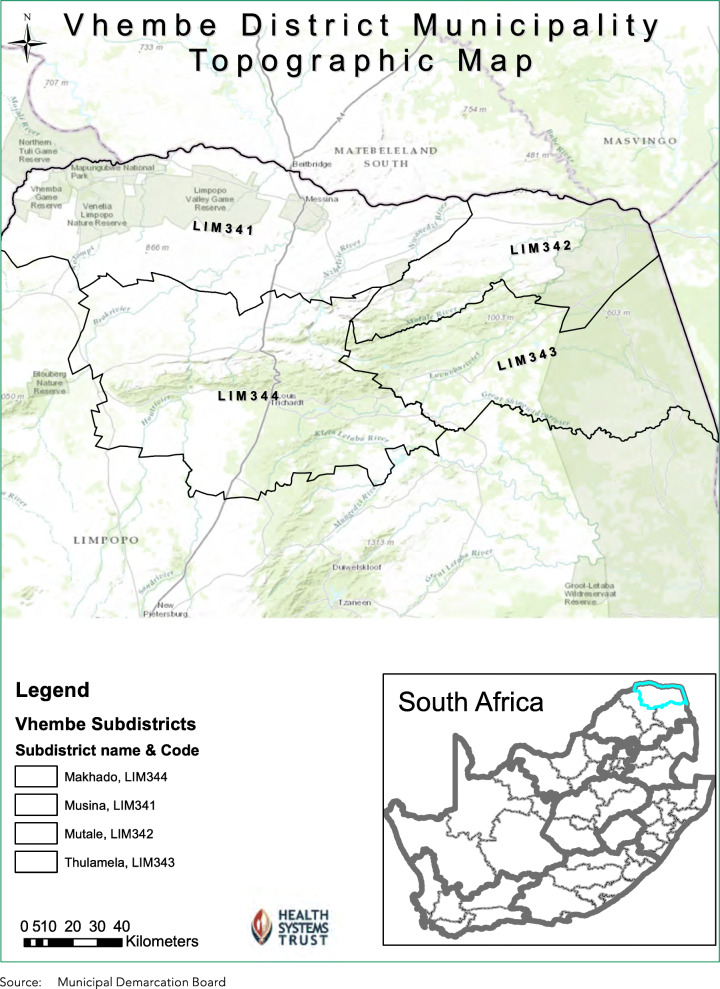
Vhembe District map indicating the four health sub-Districts [35]

The study was carried out during 2016–2017 in the Vhembe District in the northern part of the Limpopo Province in South Africa. The District covers 25,597 km^2^; have a total population of approximately 1,367,186 people and consists of four health sub-Districts namely Musina, Mutale, Thulamela and Makhado (Fig. 1). The Vhembe district is among the poorest of 52 Districts in South Africa with high levels of unemployment, and mainly rural living conditions and 93,6% of the people are uninsured and dependant on the public health sector for care [35–37].

Table 1 indicates the number of primary health care (PHC) facilities available in the Vhembe sub-Districts and the number of primary health care clinics included in this study [35–37]. The health care clinics are well distributed within the Makhado and Thulamela sub-District municipalities, while the Mutale and Musina sub-District Municipalities needs more health care facilities especially with the influx of foreign nationals through the Musina border post into the Limpopo Province. The clinics provide primary health care services through nurses and operate normally for 8 h. In addition, some nursing staff may be required to sleep at or near the health care clinic in case of emergency calls. There are 116 government owned PHC facilities in the Vhembe District Municipality from which 50 (43%) health care clinics were randomly selected for this study. Self-observation and interviews with the manager of each health care clinic during a once off visit to each of these clinics, and a standardized WHO observational checklist [38] dealing with WASH conditions, availability, supply, sanitation, hygiene and waste management aspects were used to obtain information on water, sanitation and hygiene aspects of the health care clinics. Record reviews were used to collect supplementary information on patient loads.

**Table 1:** Primary health care facilities in the Vhembe district [29–31]

sub-District Municipality	Primary health care clinics (PHC)	Community health centres	District hospitals	Regional hospital	Mobile services	PHC clinic included in study (%)
Makhado	47	4	3	0	10	18 (38%)
Thulamela	49	3	2	1	10	24 (49%)
Mutale	16	1	0	0	3	6 (38%)
Musina	4	0	1	0	1	2 (50%)
**Total number**	**116**	**8**	**6**	**1**	**24**	**50 (43%)**

### Microbiological quality assessment of water samples

All water samples were collected in sterile 250 mL water collection bottles, put on ice and taken to the laboratory to be assessed within 5 h of collection. The presence of Total coliform (TC) bacteria and *E. coli* (EC) bacteria in water samples (100 mL) from all indoor and outdoor taps at the health care clinics were assessed by the most probable number (MPN) method using the Colilert® Quanti-Tray/2000 system according to the manufacturer’s instructions (IDEXX, Westbrook, Maine, USA). Pathogenic strains of the *E. coli* positive water samples were identified using a multiplex protocol performed in a Biorad Mycycler™ Thermal cycler with specific genes as described by Omar et al., [39].

### Statistical analysis

Bacterial counts and data from questionnaires were entered on Microsoft Excel spread sheets. Descriptive analytical frequencies, percentages and 95% Confidence Intervals (CI) were used. Counts were summarised using geometric means and 95% CI for water samples that tested positive.

## Results

### General background on health care facilities

A WASH committee which oversees activities and other WASH related aspects in the clinic was present in 86% (43/50) of the health care clinics. Only 26% (13/50) of the health care clinics used the budget provided for WASH related services while only 32% (16/50) of the health care clinics had a maintenance plan available to deal with WASH related aspects in the clinic (Table 2).

**Table 2:** Presence and activities of WASH Committee/Board in the study health care clinics

Indicator	n (%)	Makhado	Thulamela	Mutale	Musina
**Committee present**	43/50 (86%)	17	19	6	1
**Do the committee have a budget?**	13/50 (26%)	7	5	1	0
**Does the committee have a maintenance plan for water facilities?**	16/50 (32%)	6	5	5	0

The health care clinics differed in size and operation (Table 3). The number of staff per clinic determined the size of the clinic and ranged between 10 and 74 staff members. Consultations included mostly out-patients who visited the health care clinics. Only four health care clinics had seen in-patients with a maximum of 10 per day. Generally, the health care clinics only allow a pregnant mother to stay for up to 6 h after giving birth. In cases of complications during or after birth, the mother and child are transferred to the nearest hospital. None of the clinics conducted any caesareans in the past 12 months. The health care clinics had no maternal or new-born deaths reported in the past 12 months.

**Table 3:** General information and statistics of the health care clinics (*n* = 50)

Indicator	Minimum	Maximum	Mean	Standard Deviation
Nr of staff at PHC facilities	10	74	24	15.34
Average number of out-patients seen by staff	50	400	124.5	71.48
Average number of in-patients seen by staff	0	10	0.36	1.56
Number of in-bed maternity bed capacity	0	22	3.68	3.80
Average number of deliveries conducted in past 1 month	0	40	6.86	7.36
Total number of deliveries in the past 12 months	0	300	92.58	80.16
Total number of caesarean sections done in the last 12 months	0	0	0	0
Total number of maternal deaths in the last 12 months	0	0	0	0

### Provision of basic water services

The health care clinics included in this study used either borehole/tube-well (40/50; 80%) or piped water into the clinic (27/50; 54%) as the main water source (Table 4). All these water points on the clinic compound were used by both patients and staff as drinking water points.

**Table 4:** Water infrastructure and practices at the health care clinics (*n* = 50)

Indicator	Total number	Vhembe sub-Districts			
		Makhado	Thulamela	Mutale	Musina
**Water source**					
Piped water into PHC facility	27	4	17	4	2
Piped water into yard/compound	1	0	1	0	0
Public stand (pipe/stand)	1	0	1	0	0
Tube well/borehole	40	17	19	3	1
Protected dug well	0	0	0	0	0
Unprotected dug well	0	0	0	0	0
Protected spring	0	0	0	0	0
Unprotected spring	0	0	0	0	0
Rainwater collection	0	0	0	0	0
Cart with small tank/drum	0	0	0	0	0
Tanker truck	0	0	0	0	0
Surface water (river/dam/lake/pond/channel)	0	0	0	0	0
**Distance of main water supply point from clinic**					
At facility (0 m)	40	15	20	5	0
Less than 200 m from clinic	4	3	0	1	0
Less than 500 m from clinic	2	0	2	0	0
1 km from clinic	1	0	0	0	1
> 1 km from clinic	2	0	2	0	0
**Other water points at clinic**	1	1	0	0	0
**Alternative option for water storage when water is not available at water points**	47	16	24	6	1
**Average queuing time at water points:**					
0 min	47	18	21	6	2
1 min	1	0	1	0	0
2 min	2	0	2	0	0

Information on the average years of construction of the water sources on the compound were not readily available during the interviews. Only 50% (25/50) of the health care clinics could provide information on the construction of the boreholes which were between 1 year and 30 years [mean: 11 years] and only 22% (11/50) of the health care clinics could provide information on the construction of the tap water pipelines which were between 2 years and 24 years [mean: 13 years]. Additionally, 94% (47/50) of the health care clinics had a water storage tank. The biggest storage tank observed was of 25,000 L in size. This assured water availability if something happened to the main water source supply. Observations indicated that on average only 32% (16/50) of the health care clinics had functional water taps. Further observations indicated that on average the distance that in-patients had to walk to the nearest tap were between 19 m and 700 m [mean distance: 20 m]. The health care clinics also kept water storage containers. Observations of theses storage containers indicated that 80% (40/50) of the health care clinics had clean water storage containers.

### Microbiological quality of available water

During the once off visit to each health care clinic, water samples were collected and assessed for Total coliform (TC) bacteria and *E. coli* (EC) bacteria counts per 100 ml water sample. In total, samples were collected from 48 inside taps and 50 outside taps from all clinics. The TC counts for both inside and outside taps ranged from 0 cfu/100 mL to > 2420 cfu/100 mL [mean TC count for inside taps: 249 cfu/100 mL; mean TC count for outside taps: 281 cfu/100 mL]. The EC counts for inside taps ranged from 0 cfu/100 mL to 649 cfu/100 mL [mean EC count: 16 cfu/100 mL]. The EC counts for outside taps ranged from 0 cfu/100 mL to 52 cfu/100 mL [mean EC count: 2 cfu/100 mL]. Only 10% (5/50) of the heath care clinics reported to be treating the water before drinking which included chlorination, filtration and receiving treated water directly from the treated municipal supply.

Table 5 indicates the health risk criteria according to the WHO [40] of all water samples collected from the inside and outside taps of the health care clinics. In this study, 62% (30/48) water samples from inside taps and 36% (18/50) outside taps had between 0 and 10 cfu/100 mL TC counts and were considered safe to use. However, 38% (18/48) inside taps and 64% (32/50) outside taps had TC counts in the unacceptable range which made them unsafe for human consumption. The water samples from 83% (40/48) inside taps 68% (34/50) outside taps had 0 cfu/100 mL for EC and was considered safe as a drinking water source. However, 24 tap water samples [17% (8/48) inside taps and 32% (16/50) outside taps] had EC counts indicating a high risk of infectious disease and unacceptable for human consumption [33].

**Table 5:** Water quality at health care facilities according to WHO risk criteria [40]

Indicator	Taps	Number of taps tested	Safe [0 cfu/100 ml]	Low risk [1–10 cfu/100 ml]	Intermediate risk [10 -100 cfu/100 ml]	High risk [**>** 100 cfu/100 ml]
Total coliform	Inside taps	48	14 (29%)	16 (33%)	8 (17%)	10 (21%)
	Outside taps	50	6 (12%)	12 (24%)	15 (30%)	17 (34%)
*E. coli*	Inside taps	48	40 (83%)	6 (13%)	2 (4%)	0
	Outside taps	50	34 (68%)	13 (26%)	3 (6%)	0

Pathogenic strains of *E. coli* were detected in all the *E. coli* positive water samples (*n* = 24) and 92% (22/24) of the water samples had more than one pathogenic *E. coli* strain (Table 6). In the outside water samples tested, the most dominant pathogenic strains included EAEC (75%), ETEC (75%) and EPEC (50%), while ETEC (100%), EPEC (88%) and EAEC (75%) strains were the most dominant pathogenic strains detected in the inside water samples tested. No EIEC strains were detected in any of the water samples.

**Table 6:** The detection of pathogenic strains of *E. coli* in the water sources used by the health care clinics

***E. coli*** pathotypes detected in water samples	Number of water samples containing this pathogenic strain	Vhembe sub-Districts			
		Makhado	Thulamela	Mutale	Musina
**Outside tap water samples**					
EAEC	75% (12/16)	5	4	2	1
ETEC	75% (12/16)	5	4	2	1
EPEC	50% (8/16)	3	3	1	1
EHEC	25% (4/16)	1	2	1	0
EIEC	0% (0/24)	0	0	0	0
**Inside tap water samples**					
ETEC	100% (8/8)	2	4	2	0
EPEC	88% (7/8)	2	3	2	0
EAEC	75% (6/8)	2	3	1	0
EHEC	38% (3/8)	0	2	1	0
EIEC	0% (0/8)	0	0	0	0

### Provision of basic sanitation services

Toilets in the health care clinics included flush toilets (49/50; 98%) and VIP toilets (41/50; 82%) with some health care clinics still using pit latrines (9/50; 18%). Toilets in the study cohort were separated for males and females [mean number of toilets per sex: 2 for males and 3 for females] and were situated either inside or outside the clinic building (Table 7).

**Table 7:** Sanitation infrastructure and practices at health care clinics (*n* = 50)

Indicator	n	Vhembe sub-Districts			
		Makhado	Thulamela	Mutale	Musina
**Main type of toilet/excreta disposal facility:**					
Flush/pour toilet to piped sewer system/septic tank etc	49	17	24	6	2
VIP toilet	41	15	20	6	0
Pit latrine with slab	8	1	7	0	0
Pit latrine without slab/open pit	0	0	0	0	0
Bucket	0	0	0	0	0
No facilities/bush/field	0	0	0	0	0
Ecosan	0	0	0	0	0
**Location of toilet**					
Separate from PHC facility	2	0	1	0	1
Within PHC facility	1	0	0	0	1
Both separated and within PHC facility	49	18	23	6	2
**The toilet facilities are constructed to accommodate people with disabilities**	47	17	24	6	0
**Sanitary condition of toilet facility at time of inspection**					
Cleanliness of seat [absence of dirt/urine/faecal matter]	27	11	11	3	2
Cleanliness of toilet hole (It is covered)	27	14	9	4	0
Cleanliness of wall [no graffiti/urine/faecal matter]	31	14	12	4	1
Cleanliness of floor [no litter/urine/faecal matter]	45	17	20	6	2
Smell [no foul odour]	22	8	12	2	0
Cleaning/cleansing materials in toilet (soap/toilet paper/sanitary tissue]	29	10	14	4	1
**How often are toilets cleaned?**					
3 or more times per day	36	15	16	4	1
1 or 2 times a day	13	2	8	2	1
Every other day	0	0	0	0	0
Once a week	0	0	0	0	0
Less than once a week	0	0	0	0	0
**Who cleans the toilets?**					
Hired cleaners	20	9	11	0	0
Patient care takers	0	0	0	0	0
health care workers	29	10	12	5	2
Community volunteers	3	0	2	1	0
**Condition of immediate area around the toilet building and entrance to the toilet**					
Grass present	38	11	19	6	2
Maintained	12	7	5	0	0
**Level of faecal matter in pit**					
full	10	2	7	1	0
half full	22	9	8	5	0
almost empty	12	5	7	0	0
n/a	6	2	2	0	2
**Is there a mechanism to empty toilets or provide an alternative if toilets are full?**	34	11	17	6	0
**Mechanism used to empty pit**					
Flush/drainage of VIP	30	10	15	5	0
Flush/drainage of VIP/Use of pit latrine	1	0	1	0	0
Never experienced full pit	7	2	4	0	1
Use sanitation in next building/section	2	1	0	1	0
VIP is used if there is no water for flush toilets	1	0	1	0	0

The toilets were reported to be cleaned between 1 to more than 3 times per day by either the health care workers (58%), hired cleaners (40%) or volunteering community members (6%). A total of 96% (48/50) of the health care clinics reported that the toilets were accessible during clinic operation hours. Observations showed that the distance the inpatients had to walk to reach the toilets ranged from 0 m to 500 m [mean distance: 12 m]. Information on the construction of the toilets were also not easy to obtain. Only 30% (15/49) health care clinics could report that the flush toilets were constructed between 5 years and 36 years ago [mean time of construction: 21 years], while only 42% (17/41) of the health care clinics could report that the VIP toilets were constructed between 1 and 30 years [mean time of construction: 8 years]. Proper lighting around the toilets were observed in 90% (45/50) of the health care clinics and 94% (47/50) of the health care clinics had proper locks and doors on the toilet cubicles for privacy and dignity. This study did not investigate the presence of waste bins in the female toilets.

### Provision of basic hygiene services

Hand washing basins with running water were observed in 90% (45/50) of the health care clinics (Table 8). The hand washing facility was either inside (25/50; 50%) the toilet facility or just outside (38/50; 76%) the toilet facility. Evidence of good hand washing practices which included the presence and availability of soap and water and signs or posters reminding patients and staff to wash hands ranged from good (31/50; 62%) to very poor (2/50; 4%) where no water and no soap and no signs or posters to remind staff and patients to wash their hands were observed in the health care clinic. Overall, signs and posters to encourage hand washing practices were only present in 64% (34/50) of the health care clinics. During the interviews, a total of 98% (49/50) of the health care clinics reported that they do give lectures on how to wash your hands to caregivers and patients.

**Table 8:** Hand washing infrastructure and practices at the health care clinics (*n* = 50)

Indicator	n	Vhembe sub-District			
		Makhado	Thulamela	Mutale	Musina
**Hand washing facilities present at PHC facility**	48	18	23	6	1
**Main type of hand washing facility**					
Wash basin and running water	45	17	21	6	0
Wash basin and buckets/small jerry cans/bottle accessed water	3	1	1	1	0
Small jerry can and water	0	0	0	0	0
Bottle with water	0	0	0	0	0
Tippy tap	1	0	1	0	0
Other	3	0	2	0	1
**Location of hand washing facility**					
Inside toilet unit	25	9	12	3	1
Immediately outside toilet facility	38	12	19	6	1
Inside PHC clinic building	2	0	2	0	0
Outside PHC clinic building	2	1	0	0	1
**Evidence of hand washing practices**					
Good [presence and availability of soap and water/patients and staff are reminded to wash hands]	31	15	13	2	1
Poor [water available/ no soap available or present/ no evidence to remind patients and staff to wash hands]	17	3	9	4	1
Very poor [no water/no soap/ no reminder to wash hands]	2	0	2	0	0

### Provision of basic health care waste management services

All the health care clinics separated the medical and solid wastes (Table 9). The distance of the solid waste disposal area from the main health care clinic building ranged between 0 and 500 m [mean distance: 61.7 m].

**Table 9:** Waste disposal infrastructure and practices in the health care clinics (*n* = 50)

Indicator	n	Vhembe sub-District			
		Makhado	Thulamela	Mutale	Musina
**Is the waste separated?**	50	18	24	6	2
**Disposal of rubbish/waste**					
Burned on/or next to facility compound	29	12	16	1	0
Buried on/next to facility compound	0	0	0	0	0
Garbage dump site on/next to facility compound	1	0	1	0	0
Transported of the facility compound	49	18	23	6	2
**Types of solid waste disposal containers provided by facility to patients/caregivers**					
Garbage container inside PHC facility only	4	3	1	0	0
Garbage container on compound only	3	1	1	0	1
Garbage container inside PHC facility and on compound	44	14	22	6	2
No garbage containers provided	0	0	0	0	0

The majority 98% (49/50) of health care clinics transported the waste of the compound and 58% (29/50) of the health care clinics burned the solid waste on the compound. None of the health care clinics buried the waste in the facility compound. The health care clinics all used the garbage site and burning method for the solid wastes when the disposal bins were full, and the waste collecting company did not collect the wastes. Additionally, 88% (44/50) of the health care clinics made sure that waste disposal bins were present on the compound as well as inside the health care clinic building. Observations showed that 72% (36/50) of the health care clinics had a clean and properly maintained compound where the grass was cut, and no litter was laying around the compound. All health care clinics reported to maintain their compound at least once a month.

## Discussion

The Sustainable Development Goal (SDG) targets for 2030 calls for the provision of affordable and quality universal health care coverage and the availability and sustainable management of water and sanitation for all [27, 41]. In South Africa, the national health policies do align with the 2030 SDG targets and prominently states the importance of health care for all her people [42–44]. The government remains accountable to provide good WASH infrastructure and should monitor and benchmark services and give training on practicing that was benchmarked in all health care facilities for good return on investment. The health care provision within South Africa is variable nationally. It is estimated that approximately 80% of the population in South Africa depend on the public health sector [45]. This increase the strain the primary health care system is taking with the statistics estimating that approximately 13,718 patients per clinic is recorded [45]. The strain includes (among other aspects) a shortage of medical doctors and nurses; infrastructure that is not maintained and poor record keeping [46–49].

A total of 80% of the health care clinics used borehole water which is classified by the WHO as an improved water source [3]. However, microbiological quality of samples taken from all the functional inside and outside taps on the clinic compound showed that many of the taps were providing water with TC and EC counts higher than WHO and South African water quality guideline counts for safe drinking water [40, 50, 51]. Other studies in developing countries globally have also shown that improved water sources at health care facilities do not necessarily guarantee safe water [6, 52–54]. A study by du Preez [55] assessing the water quality in health care facilities in the Mopani district of South Africa, who mainly used boreholes as a primary water source, showed that 55% of water samples collected from inside taps, outside taps and storage water containers had TC counts exceeding 10 cfu/100 mL and EC bacteria were found in 29% of the collected water samples. Other studies carried out in the rural areas in the Vhembe region have also showed that drinking water sources had unacceptable counts for TC and EC bacteria [56–58]. The most frequent *E. coli* pathogenic strains detected in the water samples collected from inside and outside taps on the clinic compound, included EPEC, ETEC and EAEC strains. Several studies that assessed water sources in the Vhembe District have showed the prevalence of these pathogenic strains [56, 59, 60]. Similarly, studies that screened stools in the Vhembe District have also showed that these strains are very prevalent in stools of children suffering from diarrhoea [61–63]. A study done by Kong et al., [64] estimates that EAEC causes acute and chronic diarrhoea and is a major contributor to global traveller’s diarrhoea. ETEC is one of the pathogenic agents that causes acute diarrhoea in developing countries for children under the age of five [65]. EPEC is subdivided into two types, typical (tEPEC) and atypical (aEPEC). Typical EC contains a virulence plasmid in which the bundle-forming pilus encoding (bfp) is present, while atypical EC strain does not have the bfp adherence factor [66]. In developing and developed countries, atypical EPEC is an emerging diarrheal causative agent [67] and people infected with aEPEC suffer from persistent diarrhoea [68]. During this study it was also reported from the staff that very few of the health care clinics treat the water available to the clinic. Water treatment is an aspect that should be promoted to ensure the best possible quality water and should be practiced in all the primary health care facilities in South Africa where continuous water safety is not guaranteed. There are several commercial water quality tests available globally which are inexpensive, give rapid results and are easy to operate (such as the CBT test) which the staff and caregivers can use themselves to test water samples on a regular basis and do treatment for the provision of safe drinking water [69].

The study did not evaluate actual hand washing practices of staff at the health care clinics, but it did assess the presence of hand washing basins, presence of soap and presence of posters or signs indicating or reminding staff and patients of the importance of hand washing. This study did show that washing facilities for people to wash their hands after using the toilets, were present on the inside of 72% of the toilets and just outside 50% of the toilets. Sanitation observation during the clinic visits showed that the sanitary conditions (eg cleanliness etc) were not up to standard and should be given attention to, especially with regards to cleanliness of the toilet seats. Several studies have shown that pathogenic bacterial strains do survive on toilet seats and can be a health risk to vulnerable individuals [70]. Female toilets at the health care clinics were clearly identified with signs, were separated from the male toilets and had doors with locks to ensure privacy and dignity. The sanitary conditions of toilets in all clinics should be scrutinized and improved efforts to see that hand washing practices are improved at the health care facilities, should be implemented. Several studies have showed that proper hand hygiene practices decrease the likelihood that staff will pass pathogens to and between patients during consultation and it is estimated that it can lower infections between 23 to 53% and reduce neonatal mortality by 19% [10, 71, 72]. Specific and clear visible signs and posters reminding people to practice hand hygiene, should be present at hand basins together with soap dispensers containing soap for use.

During this study it was observed that waste management policies were not always followed. Several studies have showed that improper waste management from cholera infected patients and staff can lead to faecal contamination of equipment within the health care facility and can cause fatal cholera outbreaks [73, 74]. A recent study by Oloniya et al., [75] on the efficiency of health care risk waste management in 15 rural health care facilities in the Vhembe district has noted mismanagement at different points along the waste management chain. These included poor segregation, overfilling of waste bins, inappropriate transportation and storage of wastes in sub-standard storage rooms which showed the problems currently experienced in health care facilities.

The limitations of the study included the following aspects: only 50 out of 116 health care clinics of the Vhembe District were included; the data collected through interviews with the clinic managers in this study could have been biased or not totally accurate and the study also does not offer assessment on changes of water quality or on water infrastructure repairs over time. Nevertheless, the strengths of the study included the direct systematic and objective observations by the research team which provided valuable insight into WASH related activities in health care facilities in South Africa.

## Conclusions

In conclusion, the present study has showed that several issues in WASH infrastructure and practices at health care facilities in the Vhembe district of South Africa needs improvement: water sources are not necessarily safe water sources and the water sector needs to do more to improve water quality and not just access; the sanitary conditions of toilets in all clinics should be scrutinized and upgraded; hygiene behaviour change training/workshops to improve hand washing practices at the health care facilities should be implemented and proper health risk waste management procedures should be followed. Therefore, continuous engagement and improved coordination between different ministries and stakeholders are important in order to improve WASH infrastructure and practices at health care facilities in rural areas of South Africa.

## Data Availability

The datasets used and analysed during the current study are available from the corresponding author on reasonable request.

## Abbreviations

cfu: colony forming units
CI: Confidence Intervals
*E. coli*: *Escherichia coli*
DEA: Department of Environmental Affairs
DOH: Department of Health
EAEC: Enteroadhesive *E. coli*
EC: *E. coli*
EAEC: Enteroaggregrative *E. coli*
EHEC: Enterohaemorraghic *E. coli*
EIEC: Enteroinvasisve *E. coli*
EPEC: Enteropathogenic *E. coli*
ETEC: Enterotoxigenic *E. coli*
HAI: Healthcare Associated Infections
HCRW: Health Care Risk Waste Management
km: kilometer
mL: milliliter
MPN: most probable number
mPCR: multiplex-Polymerase Chain Reaction
PHC: Public Health Care
SA: South Africa
SDG: Sustainable Development Goal
spp.: species
TC: Total coliform
WASH: Water, Sanitation and Hygiene
WHO: World Health Organisation
